# Exploring impacts of multi-year, community-based care programs for orphans and vulnerable children: A case study from Kenya

**DOI:** 10.1080/09540121.2012.729807

**Published:** 2013-06-09

**Authors:** Bruce A. Larson, Nancy Wambua, Juliana Masila, Susan Wangai, Julia Rohr, Mohamad Brooks, Malcolm Bryant

**Affiliations:** a Center for Global Health and Development and Department of International Health, Boston University, Boston, MA, USA; b Benevolent Institute for Development Initiatives, Machakos, Kenya; c Christian Aid, Nairobi, Kenya; d Center for Global Health and Development, Boston University, Boston, MA, USA

**Keywords:** orphans and vulnerable children, village savings and loan associations, food security, educational attainment

## Abstract

The Community-Based Care for Orphans and Vulnerable Children (CBCO) program operated in Kenya during 2006–2010. In Eastern Province, the program provided support to approximately 3000 orphans and vulnerable children (OVC) living in 1500 households. A primary focus of the program was to support savings and loan associations composed of OVC caregivers (typically elderly women) to improve household and OVC welfare. Cross-sectional data were collected in 2011 from 1500 randomly selected households from 3 populations: program participants (CBCO group, *n* = 500), households in the same villages as program participants but not in the program (the local-community-group = Group L, *n* = 300), and households living in nearby villages where the program did not operate (the adjacent-community-group, Group A, *n* = 700). Primary welfare outcomes evaluated are household food security, as measured by the Household Food Insecurity Access instrument, and OVC educational attainment. We compared outcomes between the CBCO and the subset of Group L not meeting program eligibility criteria (L-N) to investigate disparities within local communities. We compared outcomes between the CBCO group and the subset of Group A meeting eligibility criteria (A-E) to consider program impact. We compared outcomes between households not eligible for the program in the local and adjacent community groups (L-N and A-N) to consider if the adjacent communities are similar to the local communities. In May-June 2011, at the end of the OVC program, the majority of CBCO households continued to be severely food insecure, with rates similar to other households living in nearby communities. Participation rates in primary school are high, reflecting free primary education. Among the 18–22 year olds who were “children” during the program years, relatively few children completed secondary school across all study groups. Although the CBCO program likely provided useful services and benefits to program participants, disparities continued to exist in food security and educational outcomes between program participants and their non-OVC peers in the local community. Outcomes for CBCO households were similar to those observed for OVC households in adjacent communities.

## Introduction

The Community-Based Care for Orphans and Vulnerable Children (CBCO) program operated during 2005–2011 in Eastern Province of Kenya. The CBCO program supported the development and operation of saving and loan associations (SLAs) composed of OVC caregivers. In the final program year, the program supported 52 SLAs composed of approximately 1500 OVC caregivers and 3000 OVC (for background on SLAs, see for example Allen, 2002; Anyango, Esipisu et al. 2007). In addition to SLA activities, the CBCO program also provided direct support to these households, for example for school-related expenses (Larson & Wambua, 2011). The local community contributed several thousand days of time to the program for social-worker and extension-agent type activities (Larson & Wambua, 2011).

We completed a cross-sectional survey in May-June 2011 to document basic household- and child-level welfare indicators at the end of this program and to explore possible impacts of the CBCO program on these indicators. We present results here for household food security and basic educational attainment outcomes for school-aged children (stratified by primary and secondary school ages) and young adults (18–22), who would have been “children” during the program implementation years.

## The CBCO program in Kenya

The CBCO program used a general OVC definition consistent with criteria outlined in the Kenya Aids Indicator Survey (Republic of Kenya, Office of the President et al., 2009), the Kenyan OVC action plan (Government of Kenya, 2008), and UNICEF (United Nations Children's Fund, 2005). The vast majority of OVC caregivers in the CBCO program were women who were also heads of their households. Many were also elderly. This family structure is consistent with patterns of OVC care in other countries in sub-Saharan Africa (Beegle, Filmer, Stokes, & Tiererova, 2009).

The central component of the CBCO program was to support the development and operation of village SLAs. The SLA model implemented in the CBCO program evolved from CARE International's experience with SLAs beginning in Niger in the early 1990s (Allen, 2002; Anyango, Esipisu et al., 2007). For the CBCO program, an SLA was formed with a group of OVC caregivers (roughly 30). Through this group, the program could also provide other services to these households and their children.

In addition to loans to individuals in the SLA, SLAs typically engage in group income-generating activities (IGAs). For example, one member may have access to a piece of land that could be used for maize production, but she does not have enough labor in her household to work the land (or management skills, or seeds, etc.). With this land, the SLA as a group could purchase maize seed (perhaps hybrid maize with significantly higher yields than local varieties) and as a group allocate time to this activity. Group IGAs are a fundamental component of the CBCO SLA model. Each individual in the group does not need to be an entrepreneur and face individual financial risks, as is essentially the case with more formal micro-finance institutions.

The SLA as an institution also provides a mechanism for social support and risk pooling. If one SLA member or someone in their household becomes sick, the SLA may organize an additional voluntary contribution from SLA members to provide the member with inputs to meet their unexpected needs (e.g., cash for medicine for a sick child, some small amount of maize). In the CBCO program, these one-time activities are separate and do not show up in the SLA's accounts.

Beyond supporting the development and operation of SLAs, the CBCO program was designed to provide additional support to OVC caregivers and their children, including assistance with paying for school-related expenses. Rather than traveling to 30 individual households who might be caring for 40–60 OVC, the CBCO program worked through the SLA's regular meeting structure to meet with the 30 caregivers. Two members of each SLA group also volunteered in a social worker capacity to visit caregivers and their children at their homes (at least once per month). These volunteers were called “mentors” by the program, whose role was simply to talk to caregivers and their children (separately from caregivers for older children), listen to their concerns, and informally monitor OVC welfare.

The CBCO program included individuals trained on developing and managing SLAs, called a facilitator. The facilitator played various roles in the program. He/she supported the creation of the SLA and provided training and management support for financial matters (accounting, loan disbursement, and repayments, etc.). The facilitator was also the person the CBCO program used to distribute any materials or services to OVC caregivers and their children, usually at an SLA meeting, such as seeds for home gardens or support with school materials. And third, the facilitator acted like a rural extension agent, who assisted the SLA with learning about and identifying income-generating project ideas.

## Survey design and methods

The study was a retrospective cohort study of CBCO program participants (the intervention group) and other households living in sub-locations where the program was implemented and in nearby communities (adjacent sub-locations) where the CBCO program did not operate. Households were randomly selected from three main study groups: (1) the *CBCO group* (*n* = 500); (2) households living in the CBCO sub-locations with at least one child < 18 years of age at the time of the survey (*the* “*local community group*”*, denoted as Group L, n* = 300); and (3) households living in sub-locations adjacent to the CBCO sub-locations (*the* “*adjacent community group*”*, denoted as Group A, n* = 700).

We obtained ethics approval from the Human Ethics Research Committee of the Kenya Medical Research Institute and the IRB of the Boston University Medical Center before implementing the survey. Written informed consent was obtained from all study participants. After obtaining consent, the study questionnaire was administered verbally by a trained study enumerator. The questionnaire included sections on household demographics including education attainment, housing characteristics, asset ownership, participation in groups, and recent loan history.

The “Household Food Insecurity Access Scale (HFIAS) for Measurement of Food Access”, based on 18 questions with a 4-week recall period, was also included in the survey. The HFIAS tool has been used in several countries for measuring food security and assigning households along a continuum from food secure to severely food insecure (Coates, Swindale, & Bilinsky, 2007).

A more complete description of the study design, survey instruments, and statistical analyses is provided in the final study report (Larson and The CBCO Evaluation Team, 2012). We include in this analysis all households interviewed who reported at least one child less than 23 years of age at the time of the survey.

The final sample size used in this analysis is 1429 (CBCO Group, *n* = 486; Group A, *n* = 659; and Group L, *n*=284). Groups L and A were further stratified into two groups: those meeting the eligibility requirements for the CBCO program at the time of the survey (Group A-E and Group L-E), and those households not meeting eligibility requirements (Group A-N and L-N). A significant share of Group A and Group L households met the CBCO program eligibility criteria at the time of the survey (55% in Group A and 43% in Group L), mainly because orphaned children were living with the household.

We discuss two basic analyses here: (1) a *“disparities” analysis* and (2) a *simple impact analysis.* The disparities analysis focuses on differences between the CBCO Group and Group L-N. Comparing outcomes for the CBCO Group to Group L-N provides perspective on how “deprived” CBCO households are compared to households in their local communities whose children would not be classified as orphans or vulnerable (their non-OVC peers).

Comparing outcomes for the CBCO to Group A-E (adjacent community households meeting program eligibility requirements) is called here a “simple” impact analysis. If the adjacent sub-locations are similar to the CBCO program sub-locations (they are next door to each other with similar ecosystems and ethnicity), then A-E group could be a reasonable comparison group for discussing program impacts. We recognize that social support and public health programs, as well as economic changes, occurred throughout Eastern Province during the CBCO program years. Thus, outcomes in 2011 for all study groups incorporate any impacts of such programs. Assuming that similar programs existed or would have existed in the CBCO program areas, the simple “impact” analysis discussed in this paper is impacts beyond what might have occurred in the absence of the CBCO program (but with similar other social programs as occurred in the adjacent communities).

## Basic survey results

Table 1 summarizes basic household demographic characteristics by study group. The vast majority of CBCO households had female household heads (85%), while 56% of households in Group A-E were female headed and only 39/44% of households in Group L-N/A-N, respectively, were female headed. The highest level of formal education for any adult member of the household is modest across all study groups. For example, no adult had completed primary school or more education among 36% of CBCO households, while the majority of all households in all study groups included adults with primary school completion or less education.

**Table 1.** Household demographics.

	CBCO	A-E	L-N	A-N	L-E
Total	486	365	161	294	123
% Female-headed household	85.8	66.4	38.5	43.9	55.8
Total household size (mean)	6.1	5.8	6.1	5.7	6.3
HH size std dev	2.4	2.1	1.9	2.0	2.6
Maximum education level of any adult over 22 years of age (%)					
None	10.5	8.8	3.1	3.1	4.9
Some primary	25.5	29.1	22.4	19.1	22.0
Completed primary	26.1	25.8	28.0	36.7	28.5
Some secondary	8.9	10.7	11.2	11.2	8.1
Completed secondary	23.7	21.4	30.4	24.5	30.9
Higher	5.4	4.1	5.0	5.4	5.7

Table 2 confirms that regular participation in group-savings activities of any kind was substantially higher for CBCO group than other study groups (97% compared to 23% or less), and CBCO households also were more likely to access credit (58% compared to 20% or less).

**Table 2.** Participation in groups and borrowing.

Variable (% of total)	BID II CBCO	A-E	L-N	A-N	L-E
Participate regularly in:					
Church/religious group	20.2	15.6	21.7	14.0	14.6
Savings group	97.3	20.8	23.0	19.4	19.5
Political or advocacy group	0.8	0.8	1.2	0.3	0.8
Community service group	13.8	18.9	25.5	18.0	20.3
Income-generating group	20.8	19.5	21.1	17.4	17.1
Taken loans in the past 6 months	58.9	16.2	20.5	14.0	20.3
Took loans from:^*^					
Family member	3.1	0.3	0.6	1.0	0.0
Friend	6.8	6.3	5.0	5.1	6.5
Money lender	1.7	1.4	1.9	1.0	0.8
SLA	48.4	0.0	0.0	0.0	0.8
Cooperative/SACCO	1.4	1.6	1.9	0.3	2.4
Merry-go-round	7.2	4.1	9.9	3.7	5.7
Other type of savings group	5.4	2.2	3.1	4.1	4.1
Other	0.2	1.4	0.0	0.0	0.8

Note: ^*^Percentages are from the total in the group, with some households taking loans from multiple sources.

## Outcomes, disparities, and impact

The “HFIAS for Measurement of Food Access” has been used in several countries for measuring food security and assigning households along a continuum from food secure to severely food insecure (Coates et al., 2007). The HFIAS instrument (18 questions) was included in the survey instrument used for this study.

Educational attainment information was obtained for all children in each household interviewed. We assessed the proportion of children completing the most recent school term stratified by two age groups: 7–13 year olds (primary school age) and 14–17 year olds (secondary school age). We also assessed the proportion of children age-for-grade congruent (within 1 year plus or minus) also stratified by primary and secondary school age groups. As a third educational outcome, we assessed the proportion of young adults (18–22 years of age) who completed secondary school. These young adults would have been considered “children” at some point during the CBCO program period.

For each outcome, we compared simple proportions across study groups using OLS regression (STATA 11 with robust standard errors).

### Results – household food insecurity

The HFIA scores show poor food security for all groups in both regions (Table 3). The majority of households were classified as severely food insecure, with the exception of the L-N group (46% severely food insecure). Additional results for questions 7–9 in the HFIA instrument are included in Table 3, which highlight why they are classified as severely food insecure. Regarding continuing disparities, the mean and median HFIA scores for the CBCO group are two points higher than for the L-N group, but the differences are not substantial in magnitude or statistically significant at a 5% significance level. In terms of severe food insecurity, however, 14% more CBCO households were rated as severely food insecure as compared to the L-N group (relative risk 1.32).

**Table 3.** Food security based on HFIA instrument.

	CBCO	A-E	L-N	A-N	L-E
HFIA mean	10.82	11.47	8.70	10.57	10.30
HFIA median	11.00	12.00	9.00	11.00	10.00
	All numbers below are percentages				
*HFIA category*					
Food secure	8.02	11.54	16.77	11.90	11.38
Mildly food insecure	5.76	4.12	7.45	7.48	8.94
Moderately food insecure	25.51	23.35	29.81	26.19	23.58
Severely food insecure	60.70	60.99	45.96	54.42	56.10
*HFIA Q7: No food of any kind to eat in the household in the last 4 weeks*					
No	48.45	45.60	62.11	51.54	51.22
Rarely	21.24	23.90	18.01	21.50	22.76
Sometimes	22.89	17.58	16.15	15.36	19.51
Often	7.42	12.91	3.73	11.60	6.50
*HFIA Q8: You or household member went to bed hungry in the last 4 weeks*					
No	61.52	60.55	75.78	67.01	69.92
Rarely	19.34	22.47	14.29	18.37	13.82
Sometimes	13.79	9.86	8.70	10.88	11.38
Often	5.35	7.12	1.24	3.74	4.88
*HFIA Q9: You or household member went a whole day without eating*					
No	79.42	81.37	87.58	82.31	84.55
Rarely	9.88	7.40	7.45	8.50	5.69
Sometimes	6.79	7.95	3.73	5.78	6.50
Often	3.91	3.29	1.24	3.40	3.25

Regarding impact, the CBCO and A-E households had similar mean and median HFIA scores and essentially the same percentage of households (60%) were classified as severely food insecure in both groups. Also note in Table 3 that the L-N Group had better food security outcomes than the A-N Group, which suggests that the sub-locations used to develop the adjacent community groups might be somewhat different than the sub-locations where the CBCO program operated.

### Results – education

Responses to educational attainment of young adults and school attendance of children are shown in Table 4. Over 95% of 7–13 year olds (primary school age) completed the last school term and a large proportion (over 90%) were estimated to be age-for-grade congruent (on track) or better. No differences in these educational outcomes were observed between the CBCO, L-N, and A-E groups. For 14–17 year olds, 90% of the CBCO Group completed the previous school term (10% points below the L-N group, the same as the A-E group). Only 20–30% of 18–22 year olds had completed secondary school.

**Table 4.** Educational Outcomes.

	CBCO	A-E	L-N	A-N	L-E
Total children 7–13 years old	669	507	209	373	192
Percent of children 7–13 who completed last school term	97.31	98.03	97.61	96.51	96.35
Grade track for 7–13 year olds (%)					
Over 4 years behind	0	0.4	0.48	0.27	0
2–4 years behind	4.2	5.32	7.65	6.43	10.94
On track (±1 year)	72.52	72.97	78.95	72.65	77.6
2 + years ahead	23.27	21.31	12.92	20.64	11.46
Total children 14–17 years old	447	287	112	177	86
Percent of children 14–17 years old who completed last school term	90.38	91.64	100	96.05	93.02
Grade track for 14–17 year olds (%)					
Over 4 years behind	2.46	2.45	1.79	0	1.16
2–4 years behind	23.04	30.06	30.36	33.89	25.57
On track (±1 year)	61.3	60.49	62.5	59.32	62.79
2+ years ahead	13.2	6.99	5.36	6.77	10.47
Total number of 18–22 year olds	387	237	113	180	89
Education among 18–22 year olds					
None	0.78	0.84	0	1.11	1.12
Some primary	17.31	19.41	17.7	21.67	19.1
Completed primary	26.1	28.27	24.78	28.33	24.72
Some secondary	28.68	31.22	27.43	27.22	25.84
Completed secondary	25.06	14.77	25.66	17.78	22.47
Higher	2.07	5.49	4.42	3.89	6.74

## Conclusions

As of June 2011, CBCO households were more likely to be severely food insecure than other “non-OVC” households in their local communities, while OVC educational outcomes were close to those of their non-OVC peers. Outcomes for CBCO households were similar to those observed in households in adjacent communities who would be classified as “OVC households.”

The limitations of a cross-sectional study design to evaluate impact from a multi-dimensional, multi-year program that was not assigned randomly to program participates are well known. Given the urgency to provide and scale up services to OVC in 2006, it is not surprising that the CBCO program did not assign households with OVC randomly into treatment and control groups or sequence implementation across multiple years. With or without randomization, at least two rounds of data for at least two groups are very useful for identifying program impacts, but relevant data collected before the CBCO program began (or at least early in the program) do not exist.

Recognizing these limitations, the results presented here suggest that a low-cost SLA model may not be adequate to generate significant additional impacts on household food security and OVC educational attainment. For perspective, the cost of implementing the CBCO program at the level of implementers in each province was $49–$57 per household per year ($21–$25 per child) in 2009. The program relied on large quantities of volunteer labor, which, if valued at reasonable local wages, might increase these costs by 100%. While some variation occurred across the program years, these are very modest levels of program services. For perspective, a scaled-up “Cash Transfer Programme for Orphans and Vulnerable Children” in Kenya is reported to cost about $320 per year (see http://go.worldbank.org/2IL8VR9LX0). As another example, it costs $84 to identify one new HIV infection in a home-based HIV testing program implemented in Kenya (Negin, Wariero, Mutuo, Jan, & Pronyk, 2009).

If all the households in an SLA are essentially caught in a poverty trap, pooling resources within such households is unlikely to push them out of poverty. An SLA model within an OVC support program may make sense as a foundation for a program, but additional poverty alleviation activities (e.g., direct cash transfers, direct transfers of agricultural inputs, new jobs, etc.) are still needed.
